# Revisiting concepts of evidence in implementation science

**DOI:** 10.1186/s13012-022-01201-y

**Published:** 2022-04-12

**Authors:** Ross C. Brownson, Rachel C. Shelton, Elvin H. Geng, Russell E. Glasgow

**Affiliations:** 1 Prevention Research Center, Brown School at Washington University in St. Louis, One Brookings Drive, Campus, Box 1196, St. Louis, MO 63130 USA; 2 Department of Surgery, Division of Public Health Sciences, and Alvin J. Siteman Cancer Center, Washington University School of Medicine, Washington University in St. Louis, St. Louis, MO 63130 USA; 3 Department of Sociomedical Sciences, Columbia University Mailman School of Public Health, New York, NY 10032 USA; 4 Division of Infectious Diseases, Department of Medicine and Center for Dissemination and Implementation in the Institute for Public Health, Washington University School of Medicine, Washington University in St. Louis, St. Louis, MO 63110 USA; 5 Department of Family Medicine and Adult & Child Consortium for Health Outcomes Research and Delivery Science, University of Colorado School of Medicine, University of Colorado Anschutz Medical Campus, Aurora, CO 80045 USA

**Keywords:** Context, Equity, Evidence, Implementation science

## Abstract

**Background:**

Evidence, in multiple forms, is a foundation of implementation science. For public health and clinical practice, evidence includes the following: type 1 evidence on etiology and burden; type 2 evidence on effectiveness of interventions; and type 3: evidence on dissemination and implementation (D&I) within context. To support a vision for development and use of evidence in D&I science that is more comprehensive and equitable (particularly for type 3 evidence), this article aims to clarify concepts of evidence, summarize ongoing debates about evidence, and provide a set of recommendations and tools/resources for addressing the “how-to” in filling evidence gaps most critical to advancing implementation science.

**Main text:**

Because current conceptualizations of evidence have been relatively narrow and insufficiently characterized in our opinion, we identify and discuss challenges and debates about the uses, usefulness, and gaps in evidence for implementation science. A set of questions is proposed to assist in determining when evidence is sufficient for dissemination and implementation. Intersecting gaps include the need to (1) reconsider how the evidence base is determined, (2) improve understanding of contextual effects on implementation, (3) sharpen the focus on health equity in how we approach and build the evidence-base, (4) conduct more policy implementation research and evaluation, and (5) learn from audience and stakeholder perspectives. We offer 15 recommendations to assist in filling these gaps and describe a set of tools for enhancing the evidence most needed in implementation science.

**Conclusions:**

To address our recommendations, we see capacity as a necessary ingredient to shift the field’s approach to evidence. Capacity includes the “push” for implementation science where researchers are trained to develop and evaluate evidence which should be useful and feasible for implementers and reflect community or stakeholder priorities. Equally important, there has been inadequate training and too little emphasis on the “pull” for implementation science (e.g., training implementers, practice-based research). We suggest that funders and reviewers of research should adopt and support a more robust definition of evidence. By critically examining the evolving nature of evidence, implementation science can better fulfill its vision of facilitating widespread and equitable adoption, delivery, and sustainment of scientific advances.

For every complex problem, there is a solution that is simple, neat, and wrong. — H. L. Mencken

Contributions to the literature

- Evidence in multiple forms is a foundation of implementation science. We describe multiple types of evidence including evidence on etiology and burden, effectiveness of interventions, and implementation within context.
- We highlight what is missing in current literature on evidence and what is needed to more fully capture and characterize key evidence needed for dissemination and implementation research.
- For all types of evidence and particularly for evidence regarding dissemination and implementation, complexity and context are essential elements. We provide 15 specific recommendations to advance, specify, and broaden the field’s conceptualization and development of evidence.
- To fill the evidence gaps, we provide a set of tools and resources that begin to map out the “how-to” for accomplishing research needed to inform more equitable and sustained implementation.

## Introduction

Evidence, often informed by a complex cycle of observation, theory, and experiment [1], is a foundation of implementation science [2, 3]. Evidence is central in part because dissemination and implementation (D&I) science is based on the notion that there are practices and policies that should be widely used because scientific research concludes that they would have widespread benefits. In this context, an evidence-based intervention (EBI) is defined broadly to include programs, practices, processes, policies, and guidelines with some level of effectiveness [4]. Many of the underlying sources of evidence were originally derived from legal settings, taking on multiple forms including witness accounts, police testimony, expert opinions, and forensic science [5]. Building on these origins, evidence for public health and clinical practice comes in many forms, across three broad domains [6–8]: type 1: evidence on etiology and burden; type 2: evidence on effectiveness of interventions; type 3: evidence on implementation within context (Table 1). These three types of evidence are often not linear, but interconnected, iterative, and overlapping—they shape one another (e.g., if we have limited type 2 evidence then the ability to apply type 3 evidence is hampered). Across these three domains, we have by far the most type 1 evidence and the least type 3 evidence [6, 9].

**Table 1:** Selected terminology related to evidence and implementation science

*Type*	*Element*	*Definition*	*Sample indicators and outcomes*
Type 1 evidence: Etiology and burden	Descriptive epidemiology	The study of the occurrence of disease or other health-related characteristics in human populations, often classified under the headings of person, place, and time.	Incidence, prevalence mortality
	Burden	The impact of disease on a population	Excess risk in patients, populations, subgroups, costs
	Access	The ability to connect patients to healthcare practitioners and healthcare services	Incidence of preventable diseases, early detection, treatment
	Disparity	A particular type of health difference that is closely linked with economic, social, or environmental disadvantage	Incidence, prevalence mortality
	Etiology	The study of the causes of diseases	Effect sizes and other indicators of effect
	Social determinants and structural factors	Conditions in which people are born, grow, live, work, and age as well as the complex, interrelated social structures, and economic/political systems that shape these health outcomes and conditions	Effect sizes and other indicators of effect
Type 2 evidence: Effectiveness of interventions	Effectiveness of interventions (programs, guidelines, and policies)	Activities designed to assess, improve, maintain, promote, or modify health, health behaviors, functioning, or health conditions	Effect sizes and other indicators of effect (including heterogeneity of results)
	Effectiveness of healthcare	The study of the structure, processes, and organization of healthcare services	Performance, quality, effectiveness, efficiency, patient centeredness, equity, safety
	Practice guidelines	A standardized set of information based on scientific evidence of the effectiveness and efficiency of the best practices for addressing health issues commonly encountered in public health or clinical practice.	Recommendation (e.g., recommended, not recommended, insufficient evidence), applicability across populations and settings
	Economic evaluation	The comparative analysis of alternative courses of action in terms of both their costs and consequence (e.g., cost-effectiveness analysis)	Intervention and implementation strategy costs, cost-effectiveness ratio, return on investment, budget impact analyses, opportunity and replication costs
Type 3 evidence: Implementation and context	Context	A set of circumstances or unique factors related to the setting or community that surround a particular implementation effort	Policies, regulations, incentives, changes in priorities, setting factors, organizational characteristics, history, social, and environmental factors
	External validity	The extent to which inferences reported in one study can be applied to different populations, setting, treatments, and outcomes	Staff participation, setting participation, representativeness by geography and population, cost
	Implementation strategy	The processes or methods, techniques, activities, and resources that support the adoption, integration, and sustainment of evidence-based interventions into usual settings (e.g., ERIC taxonomy)	Acceptability, adoption, appropriateness, cost, feasibility, cost, penetration, sustainability
	Implementation mechanism	The process or event through which an implementation strategy operates to affect desired implementation outcomes	Acceptability, adoption, appropriateness, cost, feasibility, cost, penetration, sustainability
	Equity in implementation	The degree to which explicit attention is paid to the culture, history, values, and needs of the community during implementation, including any social and structural factors that may contribute to health inequities and equitable or inequitable implementation	Inequities or differences across settings or populations in acceptability, adoption, appropriateness, cost, feasibility, reach, implementation delivery/fidelity, penetration, sustainability; social determinants (e.g., living conditions, socioeconomic indicators) unintended consequences related to implementation
	Adaptation	The degree to which an evidence-based intervention is changed or modified by a user before, during, and after adoption and implementation to (a) suit the needs of the setting/local conditions; (b) respond to emerging evidence; or (c) respond to changing context	Fit with recipients, reach, data, resources, capacity, satisfaction, engagement
	Replication and transportability	The ability to transfer an evidence-based intervention to a new setting, balancing fidelity with adaptation	Acceptability, adoption, appropriateness, cost, feasibility, cost, penetration
	Scale-up	The ability to expand the coverage of successful interventions, including the financial, human, and capital resources necessary for the expansion	Usability, utility, feasibility, fidelity, adoption
	Sustainability	The ability to create structures and processes to allow an implemented EBI to be maintained and adapted in an organization or system and continue to produce benefits over time	Penetration, institutionalization, normalization, integration, capacity, infrastructure, costs, maintenance of EBI/strategy delivery, and/or continuation of health benefits

Definitions of evidence and the associated processes (how evidence is used) vary by setting. In clinical settings, evidence-based medicine is “the conscientious, explicit, and judicious use of current best evidence in making decisions about the care of individual patients” [10]. Evidence-based public health occurs across a range of community settings and is “the process of integrating science-based interventions with community preferences to improve the health of populations” [11]. Perhaps most relevant to implementation science, evidence-based decision-making is a multilevel process that involves collecting and implementing the best available evidence from research, practice, professional experience, and clinical or community partners [12–15]. A robust, equitable, and sustainable approach to evidence-based decision-making takes both challenges and strengths into account (e.g., skills, leadership priorities, resources [16–19]) and places scientific evidence and stakeholder engagement in the center of the decision-making process [20].

For all types of evidence and particularly for type 3 evidence regarding D&I, complexity and context are essential elements [21–23]. Both PCORI [24, 25] and a recent update to the MRC guidance [26] have provided statements about researching complex health interventions that provide excellent recommendations and resources. We concur with most of these recommendations and add to their points and recommendations in this article. The most effective approaches often rely on complex interventions embedded in complex systems (e.g., nonlinear, multilevel interventions) where the description of core intervention components and their relationships involve multiple settings, audiences, and approaches [26–28]. Type 3 evidence is also highly context-dependent—the context for implementation involves complex adaptive systems that form the dynamic environment(s) in which discrete interventions and interconnected implementation processes are situated [29]. For example, in models such as the Dynamic Sustainability Framework, the EBI is embedded in the context of multiple factors in a practice setting (e.g., staffing, organizational climate) which is in turn embedded in a broader ecological system with a complex set of variables (e.g., policy, regulations, population characteristics) [30]. This embeddedness also should take into account dynamism—that an EBI may stay true to its original function but need to evolve form over time to adapt to changing population needs, new evidence, and the “fit” of evidence with complex and changing context [30–32].

Much has been written about the terminology of evidence-based practice and policy. The most widely used term is “evidence-based” practice (often evidence-based medicine [33, 34] or evidence-based public health [7, 35]). Especially in Canada and Australia, the term “evidence-informed” decision-making is commonly used [15, 36]. The term “informed” is used to emphasize that public health decisions are based on research but also require consideration of individual preferences and political and organizational factors [37, 38]. Others have used the term “knowledge-based practice” or “practice-based evidence” or “practice-relevant evidence” to emphasize the importance of practice wisdom from frontline practitioners and lived experience of patients and community members [39–43]. To maximize the use of EBIs, research should inform practice and practice should inform research [44]. In our view, the most important issue is not which term to use, but rather that implementation decisions should be based on and informed by evaluation and research findings, while using rigorous methods to take into account a variety of contextual variables across multiple levels of influence (Table 2).

**Table 2:** Contextual variables for implementation across ecological levels

*Ecological level*	*Examples*
Individual	Education level Race/ethnicity/age/gender Geography/rurality Basic human needs^a^ Personal health history Readiness/motivation to undergo testing or therapy Literacy and numeracy Trust, mistrust, distrust Stigma Stress and distress Resilience Genotype and phenotype Motivation Values
Interpersonal	Family health history Support from peers Social capital Social networks Social support from family, friends, coworkers, healthcare providers
Organizational	Staff composition Staff expertise, experience, and skills Physical infrastructure Organizational and financial resources Organizational climate and culture Leadership Degree of participatory decision-making Density of organizational ties Centrality of agencies in a community Institutional racism Psychological safety Mission and priorities Guidelines and incentives Processes and procedures Training and retraining Norms Stability
Socio-cultural and community	Social norms and values Cultural norms, values, traditions Health equity History Societal stigma Community capacity, priorities, assets Local resources and investments Structural racism Shared mental models Neighborhood characteristics Access to healthcare and health promoting resources
Political and economic structures and systems	Societal values Political will Political ideology Lobbying and special interests Costs and benefits Professional guidelines Policies and regulations (both Big P and small p)

It is not anticipated that any single study would address this full list of variables; rather, this is a set of examples that can be described and narrowed via review of the literature, formative research, and stakeholder engagement

^a^Basic human needs include food, shelter, warmth, safety

Fundamental issues for implementation science involve the questions: (1) evidence on what and for whom in what settings and under what conditions? and (2) When do we have enough evidence for D&I? While the answer to this latter question will always be “it depends,” there are related questions that are useful to consider (Table 3).

**Table 3:** Determining when evidence is sufficient for dissemination and implementation

° How pressing is the health issue? ° Is there an EBI? If so, what is the quality and quantity of evidence on the EBI? ° How long will it take to develop the evidence base? ° Are there emerging or established health equity issues? ° If the study addresses social or structural determinants, might multiple health conditions benefit? ° Is the issue a priority among stakeholders? How many? Which ones? ° Are you equipped to measure a range of contextual variables? ° Are there resources to implement a study? ° Might a hybrid trial that addresses both effectiveness and implementation, be appropriate? ° Is there implementation already happening that you might evaluate? ° Is action going to be taken regardless of whether the program or policy is evidence-based or not? ° What are the consequences of not implementing? ° What are the consequences of getting it wrong?	

To facilitate the development and delivery of more equitable and sustainable interventions, we need to expand our thinking about evidence, especially for but not limited to type 3 evidence. We discuss a set of five core interrelated issues about evidence, examining (1) how the evidence base is determined, (2) context, (3) health equity, (4) policy implementation, and (5) audience/stakeholder perspectives. All areas concern some form of research or knowledge gaps in D&I science. The evidence base discussion presents a broader perspective on what is considered evidence; the context, equity, and stakeholder sections cover neglected aspects of implementation science in need of more and higher quality research; and the policy implementation section points to the need for the most pressing gaps in policy-relevant research for D&I. Across these areas, we provide a series of recommendations along with tools and resources for speeding translation of research to practice and policy.

## Selected debates about evidence

Here, we describe ongoing discussions and debates about the uses, usefulness, and gaps in evidence for implementation science, which give way to our recommendations (Table 4). While this is not an exhaustive list, it illustrates the need for more reflection and clarity across five core areas where there are major unresolved issues about evidence.

**Table 4:** Recommendations to advance evidence and implementation science

Domain	Recommendation	Rationale	Potential solutions	Actors^**a**^
*Evidence base*	1. Use an evidence typology rather than an evidence hierarchy	The choice and strength of study design is dependent on the research questions and setting, particularly the context for the study	• Identify and implement alternatives and modifications (e.g., natural experiments; interrupted time series, adaptive designs, systems modeling, mixed methods, participatory modeling, multi-level pragmatic trials, policy implementation) to the efficacy RCT • Match the research question with the study design, balanced with considerations of rigor and pragmatism	• Funders • Researchers • Policy makers^b^
	2. Increase focus on practice-based and community-defined evidence	Much of the existing evidence base is developed by university researchers in high-resource settings	• Strike a better balance between explicit (research) knowledge and tacit (lived experience) knowledge • Conduct practice-based research, particularly for low-resource settings and settings that face numerous structural and social impediments to health and well-being • Engage multi-level stakeholders and practice-based partners in substantive and meaningful ways in the context of and beyond research and research grants, including identifying stakeholder prioritized issues and outcomes	• Funders • Researchers • Practitioners
	3. Speed the pace of evidence development	The research enterprise (review processes, conducting research, publishing and disseminating research) moves slowly, often much more slowly than practice and policy	• Conduct rapid reviews/living syntheses (so-called living meta-analyses) • Use rapid methods, designs, analyses • Bring together practitioners, researchers, community members, and policy makers to identify promising innovations in need of evaluation (including realist evaluation) • Reorient funding mechanisms to be more adaptive and flexible, and to support rapid-cycle evaluation (e.g., quick addition of measures to existing studies)	• Funders • Researchers • Practitioners • Policy makers
	4. Address potential biases in implementation	Biases are often present in small scale studies that are not taken into account in larger studies or studies do not account for context	• Reconfigure small scale studies to account for generalizability biases (bias in intervention intensity, implementation support, delivery agent, target audience, duration, setting, measurement, resources required, directional conclusion, outcome) • Specify which communities, organizations, staff, and individuals are included and which are excluded and why at multiple levels and stages of a study, and their characteristics	• Researchers • Practitioners
*Context*	5. Document ways in which context drives implementation	When context is taken into account in research, study findings are more applicable to different populations, settings, and time periods	• Employ new theories, models, and frameworks (e.g., Normalization Process Theory) to understand context, including ones outside the field of implementation science that address social and community context in depth • Use mixed-methods and user-centered design approaches to study context, particularly at organizational, community, policy, and society levels • Define and apply contextual variables that lead to effective replication and may facilitate sustainability and scale-up • Investigate mechanisms of implementation strategies to enable greater generalization into different contexts	• Researchers • Practitioners
	6. Further develop pragmatic methods and measures to assess and address context	Pragmatic methods show promise by engaging multiple stakeholders, heterogeneous settings, and real-world conditions	• Make use of pragmatic measures (e.g., those that are user-friendly, sensitive to change, low cost, important to practitioners) • Apply pragmatic tools such as PRECIS-2 PS • Make use of guidelines to develop and evaluate complex interventions (e.g., the MRC guidance)	• Researchers • Practitioners
	7. Apply lessons from LMICs and other low-resource settings	There are particular challenges and opportunities for development of new evidence in LMICs	• Document and seek to replicate conditions under which innovations emerge and thrive • Apply principles of transportability research across different countries and diverse settings that have a range of capacity, resources, and infrastructure • Apply findings from task shifting research	• Funders • Researchers • Practitioners
	8. Further develop the science of adaptation	The process of modifying and refining EBIs and implementation strategies has not been well documented and understood	• Apply tools such as FRAME, FRAME IS, and other emerging coding systems to address and study key considerations in adaptation (e.g., when and how adaptations occur, whether planned or unplanned, their impact) • Use adaptation process models to guide cultural and contextual adaptations to address fit and dynamic context, while also remaining true to the original function • Better link implementation with the field of cultural adaptation to enhance the reach and equity of EBIs • Investigate ways of guiding adaptations that center on equity and investigate contexts in which EBIs may be adapted successfully versus when new EBIs may need to be developed to address specific health issues, historical experiences of populations, or sociocultural contexts	• Funders • Researchers • Practitioners
*Health equity*	9. Place greater emphasis on social determinants and structural factors that shape health inequities and inequitable implementation	Much of the evidence base is narrowly developed on diseases and risk behaviors, neglecting root and structural causes; many EBIs have not been evaluated among populations and settings experiencing inequities	• Show the value and impact of interventions that address health equity, root causes, and social determinants • Include structural racism and other equity relevant structural factors (economic inequality, stigma) in measures, frameworks, and models in assessing context and barriers/facilitators to implementation, or in planning for implementation • Map the pathways and mechanism through which upstream interventions operate to impact more proximal downstream factors and ultimately health inequities • Identify interventions that consider social context, prioritize community priorities, and build off existing community strengths/assets	• Funders • Researchers • Practitioners
	10. Integrate equity-relevant methods and measures	Equity has been under-addressed in implementation science and should be a feature of all studies	• Develop and apply models and frameworks that place a central focus on equity in both determinants and outcomes • Determine how well existing implementation strategies apply to a range of diverse populations and settings facing social and health inequities • Explicitly measure and track health equity, health inequities, and their determinants (structural racism) and how they are reduced or exacerbated by EBIs/strategies • Consider and track differential indicators of implementation (e.g., reach, feasibility, acceptability, appropriateness, adoption, implementation, sustainability) across different social groups (e.g., by race, ethnicity, age, gender, sexual orientation) or settings (e.g., urban, rural) • Prioritize equity indicators and determinants based on community and stakeholder input.	• Funders • Researchers • Practitioners
*Policy implementation*	11. Expand the scope of policy implementation research	Despite its potential impact, there are many gaps in policy implementation research	• Focus on structural interventions and community-defined interventions and policies and consider both health and social policies (that have health impacts) • Determine ways in which to build equity in all policies • Study how the meaning of evidence and processes are shaped via the interactions between policy implementation and practice change • Develop reliable and valid measures for policy implementation	• Funders • Researchers • Policy makers
	12. Apply concepts from other fields to policy implementation research	Other disciplines (e.g., political science, law, sociology) have a long history of policy research that is relevant to implementation scientists	• Apply theories from other fields to policy implementation in health • Use principles of team science to build new and vibrant transdisciplinary teams • Seek to understand the culture, norms, processes, and context of policy makers	• Researchers • Policy makers
	13. Expand knowledge of the spread of policy-relevant information	For effective dissemination of policy information, tailoring of messages and channels is needed	• Compare different messaging strategies for policy makers (e.g., social good versus cost-savings, return on investments) • Expand knowledge of the role of social media in policy implementation research (e.g., disseminating research, understanding the socio-political environment) • Expand knowledge on how to combat mis- and dis-information in policy implementation	• Researchers • Policy makers
*Audience differences*	14. Apply principles of audience segmentation and human-centered or user-centered design	Implementation research can be informed by audience segmentation principles, which were developed outside the health sector	• Select and describe characteristics of discrete audiences for dissemination and implementation • Engage community members/patients as a core audience with a commitment to return research evidence to those affected • Develop messages and channels of high salience to various stakeholders (e.g., visually appealing, brief summaries for policy makers) • Apply audience segmentation approaches from the marketing world	• Researchers • Practitioners
	15. Apply principles of framing and other communication strategies	Individuals interpret the same data in different ways depending on the mental model through which they perceive information	• Compare the effectiveness of gain versus loss framing to various audiences • Identify ways in which framing in policy advocacy can be applied to implementation science • Apply principles of narrative communication to framing to turn scientific evidence into meaningful narratives for specific audiences	• Funders • Researchers • Policy makers

^a^Individuals, groups, and community partners most likely to take action to address the recommendation

^b^Policy makers include those addressing both Big P and small p policies

### Reconsider how the evidence base is determined

The evidence base for implementation science needs to be broadened to encompass a wider range of study designs, methods, stakeholders, and outcomes. For example, the decontextualized randomized controlled efficacy trial (RCT) that attempts to control for many potential confounding factors is generally considered the gold standard for obtaining evidence on internal validity and contributing to the determination of causality of a given intervention, practice, or treatment [45]. A property of an RCT is that, with large sample sizes, it allows researchers to potentially balance known and unknown confounders. Despite the value and conceptual simplicity of the traditional efficacy RCT, its limitations have been noted [46–48]. For example, randomization may be impractical, costly, or unethical for some interventions (e.g., community-based interventions where partners have concerns about withholding a program from the community) and for many policy interventions, where the independent variable (the “exposure”) cannot be randomized. Tools such as PRECIS-2 and the newer PRECIS-2 PS help enhance the real-world utility of RCTs (pragmatic trials) [49, 50]. For some settings and interventions, alternative and more rapid-cycle and adaptive designs are needed to elucidate effects including quasi-experiments, observational trials, iterative assessments and actions, natural experiments, and mixed-methods studies [51–55]. Often in implementation science what we want to know is how one strategy adds to a range of strategies already being delivered within an existing environment a concept called “mosaic effectiveness” [56].

For clinical and public health practice, the generalizability of an EBI’s effectiveness from one population and setting to another (and ideally across a diverse range of populations and settings)—the core concept of external validity—is an essential ingredient. Systematic review and practice guidelines, which are often the basis for an implementation study, are mainly focused on whether an intervention is effective on average (internal validity) and have commonly given limited attention to specifying conditions (settings, populations, circumstances) under which a program is and is not effective [57–59]. For implementation science, there are many considerations and layers to the notion of whether an evidence-based practice applies in a particular setting or population [59]. Tools such as ADAPT [60] or process models like ADAPT-ITT [61] can be useful in transferring EBIs from one setting to another while taking contextual variables into account. Models such as FRAME and FRAME-IS are helpful for tracking and building the evidence base around what types of adaptations are associated with improved or decreased effectiveness or implementation outcomes (and for which settings and populations?) [62, 63].

The question of whether an EBI applies involves a set of scientific considerations that may differ from simply knowing average treatment effects. These include balancing of fidelity to the original EBI functions with adaptations needed for replication and scale-up [64], as well as considerations as to when there may be a need to “start from scratch” in developing a new intervention as opposed to refining or adapting an existing one (e.g., when the nature of the evidence for an EBI does not fit the sociocultural or community context). There is a pressing need for research on the strengths and limitations of practitioner-driven and community-centered adaptation of EBIs, which is likely to enhance relevance, feasibility, and sociocultural appropriateness and acceptability, as well as fit with implementation context [65–67]. There are also potential considerations when adapting EBIs or implementation strategies (e.g., costs, resources needed, potential reduction in effectiveness) [63, 68, 69]. It has also been suggested that a greater emphasis is needed on both the functions of an intervention (its basic purposes, underlying theoretical premise) and forms (the strategies and approaches used to meet each intervention function) [64], opening the door to inquiry about how fidelity to function may demand adaptations (or in some cases transformation or evolution) in form.

Additional evidence is needed on the inter-related concepts of null (ineffective) interventions, de-implementation, and mis-implementation [70–72]. From null intervention results, we can learn which parts of an EBI or implementation strategy need to be refined, adapted, or re-invented. Data on null interventions also informs for whom and under what conditions an EBI or implementation strategy is “evidence-based.” De-implementation is the process of stopping or abandoning practices that are not proved to be effective or are possibly harmful [73], whereas mis-implementation involves one or both of two processes: the discontinuation of effective programs and the continuation of ineffective practices in public health settings [70]. Many of the contextual variables in Table 2 strongly affect de-implementation and mis-implementation.

Emerging perspectives in data science and causal inference may help advance type 3 evidence. If contextual heterogeneity is the norm, then the scientific task in any one study population is to produce data that address relevance across diverse external settings. Useful methods to do so are becoming available and suggest that the more we know about mediators/mechanisms and modifiers of effects in implementation, the more interpretable findings could be in different settings and populations [74–76]. For example, consider the question of whether evidence for audit and feedback on the use of EBIs in HIV clinics from randomized trials in Boston could apply to HIV clinics in Nairobi, Kenya. Let us assume that in Boston, researchers learn that the credibility of the data is a key driver of successful implementation (e.g., clinicians who doubt the veracity of metrics from the electronic health record are less likely to respond). Given the widespread challenges of data accuracy in the nascent electronic health records in this specific setting in Africa (and extensive literature documenting this challenge), audit and feedback as an implementation strategy can be anticipated to have limited implementation relevance as well as effectiveness. Using data from Boston to infer (in this case that it might not work) in Nairobi depends on knowing critical mediators of audit and feedback in Boston (i.e., the credibility of data on provider performance). In some situations, a completely different implementation strategy may be needed that is better suited to local conditions. One further implication is that this directs research efforts to not only find effects in Boston, but how they came about (type 3 evidence).

### Improve understanding of contextual effects on implementation

The complexity and dynamic nature of implementation necessitate continual attention to context (i.e., active and unique factors that surround implementation and sustainability [77, 78]) [22, 79, 80]. When context is taken into account in research, the study findings are more likely to indicate the conditions under which evidence does or does not generalize to different populations, settings, and time periods [23]—yet too often context is inadequately described or not fully elucidated [81]. Contextual conditions also drive and inform the adaptation of EBIs to populations and settings that differ from those in which it originally developed [82]. It is useful to consider contextual issues of relevance for implementation across levels of a socio-ecological framework (individual, interpersonal, organizational, community, policy) (Table 2) [79].

The challenging scientific task of “unpacking” context requires three activities. First, contextual effects in any study setting or across settings and/or systems should be enumerated (e.g., a set of variables in Table 2). Second, since one cannot measure everything, part of building the evidence base involves determining which aspects of context are most salient for implementation within and across settings. Third, implementation research should also seek to measure the presence, distribution, and intensity of those contextual factors in target settings in which a research study is not being undertaken, but where one might want to apply evidence.

Within an implementation research project, context is dynamic and should be assessed across all stages of a study [83]. Too often, dynamic contexts are not fully understood or assessed [30]. In some cases, the context for delivery (e.g., a particular clinical setting) is relatively stable, but the target of the intervention (e.g., a particular pathophysiology; guidelines for cancer screening) is dynamic and emergent. In a more complex intervention trial, both context and targets are dynamic and emergent [22, 84].

During implementation planning, a needs and assets assessment (formative research) should account for historical, cultural, social, and system factors that may shape implementation and the implementation climate, including forms of structural or institutional racism (e.g., inequitable practices and policies), medical mistrust, institutional and providers’ biases and norms that may create or reinforce biases or inequities, as well as community strengths and assets that may inform implementation efforts. Tools such as critical ethnography can be useful during needs assessment to understand interactions between the ensembles of actors, agencies, interventions, and other contextual variables [85]. When selecting EBIs to be tested in an implementation study, context may affect both internal validity and external validity. Systematic reviews, which are often the source of EBIs, use a relatively narrow hierarchy of evidence [86] and tend to strip out implementation context when trying to make a summary (often quantitative) judgement about the average effectiveness of an EBI (e.g. for most populations and settings). For many settings in which we are conducting implementation studies (e.g., lower- and middle-income countries [87]), we may not have a strong evidence base, guidelines, or interventions that have been tested through “gold-standard” RCTs and if they have, they are often not under conditions similar to those in which the EBI will now be applied.

Context in global settings presents unique considerations, particularly in lower- and middle-income countries (LMICs) and other settings that have limited resources and face numerous structural barriers to health (e.g., in the USA, federally qualified health centers, donor-funded vertical health programs in lower- and middle-income countries). Among the considerations is the relevant evidence base for implementation—when settings vary tremendously, particularly the social and political context and systems/organizational infrastructure: Do researchers and implementers need to start anew in building the evidence base for implementation, answering many of the questions in Table 3? There is some evidence that in settings with constrained resources, intervention and methods innovations may be fostered due to the need for creativity and adaptations (e.g., task shifting [88]) when choices are restricted [89]). Adaptive designs (where interventions and strategies are modified in response to emerging data) may be particularly useful in LMICs since they may allow a team to begin with low-intensity/low-resource approaches, and refine or intensify as needed [90–92].

Transportability theory has been applied to assess whether findings about the effects of an implementation strategy in one setting can be used to infer in another, and if so, whether it is likely to work [93]. Context, when defined narrowly as the causes of an outcome that differ from one setting to another, asks science to focus on two measurement tasks. In the initial context where a strategy is being tested, it will be important to measure the steps that mediate or moderate the effects of the strategy on the outcome as well as factors that influence those steps. Hypotheses not only about effects but also about how and why they occur across diverse settings are important to inform the measurement architecture.

Context is also important during the process of broader dissemination of evidence-based approaches. There is a well-documented disconnect between how researchers disseminate their findings (including EBIs) and how practitioners and policy makers learn about the latest evidence [14]. Applying principles of designing for dissemination (D4D) allows researchers to better account for the needs, assets, priorities, and time frames of potential adopters and stakeholders [94**, **95]. An active D4D process emphasizes the design phase of an implementation research project. A D4D process anticipates dissemination of products (e.g., an evidence-based implementation strategy) by developing a dissemination plan that takes into account audience differences, product messaging, channels, and packaging [96]. In the future, this proactive D4D process could usefully more fully address designing for equity and sustainment, as well as dissemination.

### Sharpen the focus on health equity

**Fig. 1:**
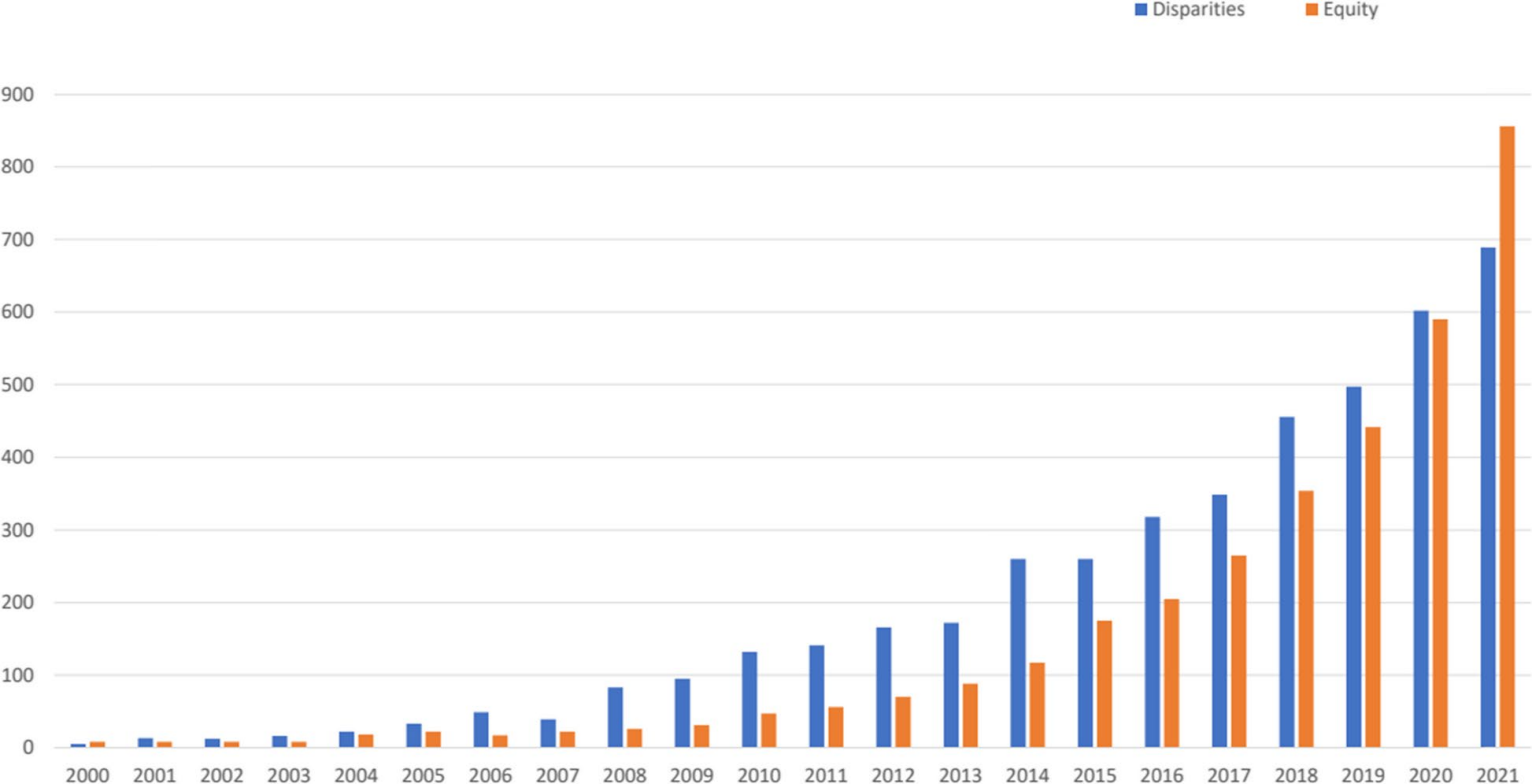
Number of annual publications on health disparities and health equity

Addressing heath disparities and promoting health equity is becoming a more central and explicit focus of implementation science [92, 97–102]. Health equity is a framing that shifts from a deficits approach (disparities) to one focused on what society can achieve (equity) [103]. An equity focus also recognizes the unjust nature of inequities, naming root/structural causes [104]. This emphasis is documented in publication trends over the past two decades. Figure 1 shows trends of publications from January 1, 2000, to December 31, 2021, using two search strings in PubMed: 1) “health disparities” AND [“implementation science” OR “implementation research” or “knowledge translation”] and 2) “health equity” AND [“implementation science” OR “implementation research” or “knowledge translation”]. For most of the past two decades, research has been framed more often with a disparities focus than with an equity focus—disparity publications were two- to three-fold more common than equity articles from 2006 to 2014. However, in 2021, the number of equity-framed publications greatly exceeded the number of disparities-framed publications.

To move towards the goal of achieving health equity, it is critical that implementation science expands the quantity, quality, and types of evidence produced and prioritized, as well as who and what settings are (1) reflected in that evidence (representativeness) and (2) involved in its generation and interpretation (representation). For many health conditions and populations, we have adequate descriptive (type 1) data that can guide what to address (e.g., the size and nature of disparities). However, we often lack sufficient data on EBIs and strategies that are effective in reducing inequities and/or promoting equity [92]. Often, available EBIs inadequately address or account for many relevant social, cultural, structural, and contextual conditions that shape both health inequities and have implications for EBI implementation [92, 105, 106]. There are challenges in generating evidence on inequities, including potentially smaller sample sizes across various social dimensions through which inequities exist, which may limit subgroup heterogeneity analyses (e.g., by race or ethnicity) [107, 108] (see Table 2). As we build the evidence base of EBIs to actively promote equity, there is a need to understand the core elements of equity-focused interventions and strategies, and to do so for the range of social dimensions through which health inequities may exist (e.g., race, immigration status, gender, sexual orientation, location) and their intersection [109].

A foundational challenge here is that many EBIs were not developed with or tested among settings or populations that experience inequities or with the goal of promoting health equity and may unintentionally contribute to or exacerbate inequities [110–112]. This results in part from the reductionist way in which EBIs are often developed, deployed (a linear, “cause and effect” approach), and tested [113], paying inadequate attention to the complex and interrelated social determinants of health and root causes of health inequities (e.g., structural racism, inequitable allocation of resources and opportunities) [114–118].

We need to engage a wider range of partners from lower resource settings earlier and throughout the research process and in meaningful ways to build a broader and more relevant array of equity-focused EBIs that are feasible, acceptable, culturally appropriate, and address root causes. We also need to expand what we “count” as EBIs in public health and clinical research, broadening the focus from a narrower view of individual, interpersonal, and organizational interventions, to also include community, policy, and multi-sector interventions that have the potential to make larger shifts in health inequities. Such broadening of evidence with an eye towards health equity will consider moving beyond a more singular focus on our EBI repositories and including and evaluating existing promising community-defined evidence and interventions [92, 119, 120]. In expanding the evidence-base with the goal of promoting health equity, there are significant opportunities to develop and deploy EBIs in sectors outside of health (e.g., schools, workplaces, social services agencies, juvenile justice settings) where in many cases, the reach and impact can be greater than in the health sector [121]. Additionally, as we expand this evidence base, it may be beneficial to prioritize development and evaluation of interventions, practices, and policies that can reduce underlying structural and social factors (e.g., structural racism) and their downstream effects on health inequities [120].

Equity should be a core criterion for valuing evidence. This value statement should be reflected in priorities of funders, how research questions are framed, how research resources and decision-making are distributed, and how studies are conducted, evaluated, and reviewed. Implementation science has a role in recognizing that a negative consequence of our social and economic systems is the concentration of resources and health. These systems create inequities, so when thinking about closing an implementation gap, we should recognize the context—that such a gap is often an outgrowth of these systems and must be addressed and transformed. Equity needs to be prioritized and made more explicit as part of engagement efforts, which includes consideration of power imbalances (who is and is not involved in making key decisions) and timing of when and how partners are engaged (e.g., who is involved in EBI development and deployment, how communities are reflected in co-creating the evidence) [95, 120]. Reflection questions and step-by-step guidance can help guide study planning with an equity focus [102, 120].

### Conduct more policy implementation research and evaluation

Health and social policies, in the form of laws, regulations, organizational practices, and funding priorities, have a substantial impact on the health and well-being of populations and create the conditions under which people can be healthy and thrive- or not [122, 123]. Clinical and public health guidelines inform policy implementation by providing the basis for legislation, informing covered services in health plans, and advancing policies that support health equity [124–128]. Policies often address the underlying social and structural conditions that shape health and inequities—this in turn provides opportunities for policy implementation to frame accountability for organizations and systems.

Policy implementation research, which has been conducted since the 1970s across multiple disciplines [129, 130], seeks to understand the complexities of the policy process and increase the likelihood that evidence reaches policymakers and influences their decisions so that the population health benefits of scientific progress are maximized [131]. A key objective of policy implementation research is the enactment, enforcement, and evaluation of evidence-based policies to (1) understand approaches to enhance the likelihood of policy adoption (process); (2) identify specific policy elements likely to be effective (content); and (3) document the potential impact of policy (outcomes) [132]. Especially in the USA, policy implementation research is underdeveloped compared to other areas in implementation science. For example, a content analysis of all projects funded by the US National Institutes of Health through implementation research program announcements found that only 8% of funded grants were on policy implementation researc h[133]. Few of these studies had an explicit focus on equity or social determinants of health.

Policy researchers have utilized a variety of designs, methods, and data sources to investigate the development processes, content, and outcomes of policies. Much more evidence is needed, including which policies work and which do not (for what outcomes, settings, and populations), how policies should be developed and implemented, unintended consequences of policies, and the best ways to combine quantitative and qualitative methods for evaluation of “upstream” factors that have important implications for health equity [134]. There is also a pressing need for reliable and valid measures of policy implementation processes [135]. These knowledge gaps are unlikely to be addressed by randomized designs and are more likely to be addressed using quasi-experimental designs, natural experiments, stakeholder-driven adaptations, systems science methods, citizen science, and participatory approaches [51, 66, 136–139].

Several other areas in policy implementation research need attention. First, policy makers often need information on a much shorter time frame than researchers can deliver—this calls for the use of tools such as rapid-cycle research [140] and rapid realist reviews [141]. Second, we need to better understand the spread of policies, including the reasons that ineffective policies spread [142], the role of social media [131], and ways to address mis- and dis-information in the policy process [143]. Finally, more emphasis is needed on the reciprocal, often horizontal, interactions between organizations and the development of policy-relevant evidence [144]. For this inter-organizational research, the role of policy intermediaries (those who work in between existing systems to achieve a policy goal) has gained attention due to their critical roles in policy implementation research [145]. Strategies and tools to address several of these issues are provided in recent reviews [146, 147] and in Table 4.

### Pay greater attention to audience and stakeholder differences

There are multiple audiences of relevance for developing, applying, disseminating, and sustaining the evidence for implementation science [148]. When seeking effective methods to generate, implement, and sustain EBIs, it is important to take into account the characteristics of each audience and stakeholder group, what they value, how to balance different viewpoints, and how to combine stakeholders’ experience and research evidence. Across these stakeholder groups, research evidence is only one of many influential factors influencing adoption, implementation, and sustainment of EBI [6, 15, 40].

Key audience categories include researchers, practitioners, and policy makers (Table 5). Researchers are one core audience. These individuals typically have specialized training and may devote an entire career studying a particular health issue. Another audience includes clinical and public health practitioners who seek practical information on the scope and quality of evidence for a range of EBIs and implementation strategies that are relevant in their setting. Practitioners in clinical settings (e.g., nurses, physicians) have specialized and standardized training whereas the training for public health practitioners is highly variable (most public health practitioners lack a public health degree [149]). A third group is policy makers at local, regional, state, national, and international levels. These individuals are faced with macro-level decisions on how to allocate public resources. Policy makers seek out distributional consequences (i.e., who has to pay, how much, and who benefits) [150] and in many policy settings, anecdotes are prioritized over empirical data [9]. The category of policy makers also includes funders—these funders may be elected officials and “small p” policy makers (organizational leaders) who make funding decisions within their settings.

**Table 5:** Differences in evidence-related characteristics and needs among audiences

*Characteristic*	*Researcher*	*Practitioner (clinical, public health)*	*Policy maker*^*a*^
*Time in position*	Longer	Middle to longer	Shorter
*Training*	Specialized	Specialized for some, but generalized for others	Generalized
*Personal connection to constituents*	Low	Moderate to high	Moderate to high
*Knowledge span*	Deeper knowledge on a small number of issues	Moderate knowledge on wide set of issues (often more specialized in larger agencies)	Less depth, wider breadth
*Decisio-making based on external factors*^b^	Low	Moderate	High
*Time spent on a particular issue*	Longer	Moderate	Shorter
*Role in the evidence development process*	Generation, synthesis, publication, implementation, dissemination	Planning, evaluation, implementation, dissemination, sustainment	Adoption, implementation, dissemination, sustainment, funding
*Primary types of evidence relied upon*	Science, evidence reviews, experimental experience from the field, general evidence	Science, evidence reviews, real-world experience from the field, personal experience, local evidence	Real-world stories, constituents, gatekeepers, party priorities, media, science, policy briefs
*Barriers to the use of evidence*	Time, predominant focus on RCTs, lack of attention to context, slow speed of research	Time, lack of access to peer-reviewed evidence, lack of incentives, low priority of leadership, perceived lack of relevance, competing demands	Time, lack of interest, complexity of evidence, new demands, rapidly changing context

^a^Policy makers include funders of research

^b^External factors commonly include habit, stereotypes, and cultural norms

The relevance and usefulness of evidence vary by stakeholder type (Table 5) [151]. Research usefulness can be informed by audience segmentation, where a product promotion strategy is targeted to the characteristics of a desired segment—a widely accepted principle in marketing [152]. Audience segmentation can be informed by the process of user-centered design and decision-centered processes, in which the product (e.g., an implementation strategy) is guided in a systematic way by the end-users of the product [153–155].

Framing is another important factor in considering audiences for D&I. Individuals interpret the same data in different ways depending on the mental model through which they perceive information [156]. For example, policy makers often perceive risks and benefits not in scientific terms but in relation to (usually short term) emotional, moral, financial, or political frameworks [157, 158]. In practical terms for implementation science, framing for a particular health issue for a community member or patient might relate to the ability to raise healthy children whereas framing for a policy maker might relate to cost savings from action or inaction. Cost and economic evaluation are key considerations for a range of stakeholders involved in implementation, yet too often the perspectives of diverse stakeholders are not well considered, acted upon, or reported [159].

## Next steps for addressing gaps

The “how-to” for broadening the evidence base for implementation science will require several actions. First, we need to prioritize the evidence gaps and possible ways of filling these gaps—many ideas are shown in Table 4. Next, resources and tools are needed to address evidence deficits (Table 6). All tools listed are available free of charge and provide enough background and instructions to make them useful for a wide range of users—from beginners to experts. The tools listed cover multiple, overlapping domains: (1) engagement and partnerships; (2) study planning; (3) research proposals, articles, reporting, and guidelines; (4) and dissemination, scale-up, and sustainability. In addition to the resources in Table 6, there are many other portals that provide valuable information and resources for implementation research across multiple domains (e.g., technical assistance, mentorship, conferences, archived slides, webinars) [160–168].

**Table 6:** Selected resources and tools to support practice and research on evidence-based dissemination and implementation

*Category*	*Name*	*Description*	*Weblink*
*Engagement and partnerships*	Community Tool Box	The Community Tool Box is a free, online resource for those working to build healthier communities and bring about social change. The Tool Box seeks to promote community health and development by connecting people, ideas, and resources.	https://ctb.ku.edu/en
	Engage for Equity	The tools provide a step-by-step approach for research partnerships to examine where they are now and where they want to be in the future. Each step includes a short description and an interactive exercise or tool.	https://engageforequity.org/tool_kit/
	Advancing Health Equity Toolkit	This practice-oriented toolkit leads agencies, teams, community-based organizations, and community partnerships through different public health processes using a health equity lens. The modules include interactive reflection questions across a framework for evidence-based decision-making.	Home | Evidence-Based Decision Making & Health Equity (wixsite.com)
	Stakeholder Engagement Navigator	The Navigator is designed to help teams select the most appropriate engagement method or tool for a particular project. It is an interactive tool that takes into account the purpose, resources, frequency of engagement, and expertise.	https://dicemethods.org/Tool
*Study planning*	Dissemination and Implementation Models in Health Research and Practice	An interactive, online resource designed to help researchers and practitioners navigate dissemination and implementation theories, models, and frameworks through planning, selecting, combining, adapting, using, and linking to measures. Newly added frameworks address the interface between health equity and implementation science.	https://dissemination-implementation.org/
	T-CaST (Theory, Model, and Framework Comparison and Selection Tool)	T-CaST offers explicit criteria to facilitate theory comparison during the selection process. The tool is also potentially useful in selecting theories, models, and framework beyond the field of implementation science.	https://impsci.tracs.unc.edu/tcast/
	PRECIS-2 (PRagmatic Explanatory Continuum Indicator Summary) and PRECIS-2 PS	PRECIS-2 is a tool to help in designing health services research and to consider where a trial lies across 9 dimensions across the pragmatic/explanatory (efficacy) continuum; the newer PRECIS-2 PS is focused on designs related to provider strategies for implementation studies.	https://www.precis-2.org/ https://implementationscience.biomedcentral.com/articles/10.1186/s13012-020-01075-y
	APEASE (Acceptability, Practicability, Effectiveness, Affordability, Side-effects, and Equity)	The APEASE criteria provide a framework for assessing interventions, intervention components, and ideas. APEASE can be applied to anything from a general concept to a detailed plan for a proposed intervention, or a formal evaluation of an intervention that has already been implemented.	https://assets.publishing.service.gov.uk/government/uploads/system/uploads/attachment_data/file/875385/PHEBI_Achieving_Behaviour_Change_Local_Government.pdf
	MOST (Multiphase Optimization Strategy)	MOST is a research framework, based on engineering principles, for determining the most efficient and effective version of an intervention. It uses a 3-phase approach to assess the effectiveness of individual program elements and consider whether effectiveness varies depending on context.	https://www.hvresearch.org/precision-home-visiting/innovative-methods/multiphase-optimization-strategy-most/
	The Hexagon Tool	At any stage of implementation, the Hexagon Tool can be used by communities and organizations to better understand how a new or existing program or practice fits into an implementing site’s existing work and context.	https://nirn.fpg.unc.edu/resources/hexagon-exploration-tool
	Annotated Bibliography of Economic Analysis Resources for Implementation Science	This tool is a compilation of resources, tools, and studies about cost/cost-effectiveness research in implementation science. It covers costing methods and cost-effectiveness analyses that are important for measuring and improving the value of healthcare and public health practices.	cost-annoat-biblio-disc-one-pager-3122119e99fe6302864d9a5bfff0a001ce385.pdf (cuanschutz.edu)
	Measuring Health Policy Implementation	This website is designed to help policy researchers, evaluators, and implementation science researchers identify and select measures to assess the implementation of health policies in a variety of settings (e.g., hospitals, outpatient clinics, neighborhoods, schools).	https://www.health-policy-measures.org/
*Research proposals, articles, reporting, and guidelines*	Tool for Rating Research Proposals for Sensitivity to Health Equity Issues	This tool assesses research proposals for their sensitivity to health equity issues. The tool consists of a series of questions that prompt for evaluation of how well equity issues have been considered in terms of the population context, study rationale, intervention design, sample design, data collection and analysis plan, evidence of community engagement, and team composition.	https://ajph.aphapublications.org/doi/suppl/10.2105/AJPH.2019.305221
	GRADE (Grading of Recommendations, Assessment, Development and Evaluations)	GRADE is a transparent framework for developing and presenting summaries of evidence and provides a systematic approach for making clinical practice recommendations.	https://bestpractice.bmj.com/info/us/toolkit/learn-ebm/what-is-grade/
	Expanded CONSORT (Consolidated Standards of Reporting Trials)	The expanded CONSORT includes data about participation and representativeness at multiple levels of settings, as well as staff and individual recipients, and about intervention sustainability after project support ends. It adds a focus on transparent reporting of inclusions, exclusions, and participation at multiple levels and includes a fillable PDF for manuscript submissions.	https://www.re-aim.org/expanded-consort-figure-for-planning-and-reporting-d-i-research/
	Standards for Reporting Implementation Studies (StaRI) Statement	StaRI is used for reporting of implementation studies, which employ a range of study designs to develop and evaluate implementation strategies with the aim of enhancing adoption and sustainability of effective interventions	https://www.equator-network.org/reporting-guidelines/stari-statement/
*Dissemination, scale-up, and sustainability*	Dissemination Planning Tool	A tool to help researchers evaluate their research and develop appropriate dissemination plans, if the research is determined to have “real-world” impact	https://www.ahrq.gov/patient-safety/resources/advances/vol4/planning.html
	ExpandNet	A global network of representatives from international organizations, non-governmental organizations, academic and research institutions, ministries of health, and specific projects who seek to advance the science and practice of scaling up	https://expandnet.net/
	Clinical Assessment Sustainability Tool (CSAT)	**The CSAT** measures the sustainability of evidence-based practices in clinical settings. Users receive a tailored report that can be used by clinical and healthcare settings to plan for and implement changes within their organization.	https://www.sustaintool.org/csat/
	Program Assessment Sustainability Tool (PSAT)	**The PSAT** measures the sustainability of evidence-based practices in community settings. Users receive a tailored report that can be used by public health and community organizations to plan for and implement changes within their organization.	https://www.sustaintool.org/psat/

This table is illustrative and is not meant to be comprehensive. We have focused on sources that are more regularly updated

Capacity is a core element for building a stronger, more comprehensive, and equitable evidence base. Capacity can be developed in multiple ways, including supporting the “push” for implementation science where researchers are trained to develop the evidence for implementation and skills in evaluation. Evaluation skill building should take into account the principles of realist evaluation, a mixed-methods approach that takes into account multiple contextual variables [169]. There is a significant number of implementation science training opportunities across countries [160, 170, 171], though few have an explicit focus on many of the issues we have highlighted (e.g., health equity, designing for dissemination, sustainability, policy implementation). There has also been inadequate training and too little emphasis on the “pull” for implementation science (e.g., training the practitioners/implementers) [170, 172]. This emphasis on “pull” should embrace the audience differences in Table 5. There is even less evidence on who and how to conduct capacity building, especially in low-resource settings [171, 173].

There are also macro-level actions that would facilitate a broader and more robust evidence base. For example, funders and guideline developers should adopt a more comprehensive definition of evidence, addressing many of the recommendations outlined in Table 4 and above. This could include an alternative or addition to GRADE, incorporating methods of appraising research that does not automatically elevate RCTs (particularly when answering policy-related research questions). Similarly, it is helpful for study sections to be oriented to a wide array of evidence, particularly type 3 evidence. This will require some learning as well as some unlearning—as an example, we need to broaden our understanding of contextual mediators and moderators of implementation, which are likely to vary from those identified in highly controlled experiments.

## Conclusion

Over the past few decades, there has been substantial progress in defining evidence for clinical and public health practice, identifying evidence gaps, and making initial progress in filling certain gaps. Yet to solve the health challenges facing society, we need new and expanded thinking about evidence and commitment to context-based decision-making. This process begins with evidence—a foundation of implementation science. By critically examining and broadening current concepts of evidence, implementation science can better fulfill its vision of providing an explicit response to decades of scientific progress that has not translated into equitable and sustained improvements in population health [92].

## Data Availability

Not applicable.

## Abbreviations

APEASE: Acceptability, Practicability, Effectiveness, Affordability, Side-effects, and Equity
CONSORT: Consolidated Standards of Reporting Trials
D4D: Designing for dissemination
EBI: Evidence-based intervention
ERIC: Expert Recommendations for Implementing Change
FRAME: Framework for Reporting Adaptations and Modifications-Enhanced
FRAME-IS: Framework for Reporting Adaptations and Modifications to Evidence-based Implementation Strategies
GRADE: Grading Recommendations Assessment and Development Evidence
HIV: Human immunodeficiency virus
LMICs: Lower- and middle-income countries
MOST: Multiphase Optimization Strategy
MRC: UK Medical Research Council
PRECIS-2: PRagmatic Explanatory Continuum Indicator Summary
PRECIS-2 PS: PRagmatic Explanatory Continuum Indicator Summary for providers
RCT: Randomized controlled trial
StaRI: Standards for Reporting Implementation Studies
TCaST: Theory, Model, and Framework Comparison and Selection Tool
